# The Risk Factors, Incidence and Prognosis of Postpartum Breast Cancer: A Nationwide Study by the SMARTSHIP Group

**DOI:** 10.3389/fonc.2022.889433

**Published:** 2022-07-01

**Authors:** Sungmin Park, Ji Sung Lee, Jae Sun Yoon, Nam Hyoung Kim, Seho Park, Hyun Jo Youn, Jong Won Lee, Jung Eun Lee, Jihyoun Lee, Ho Hur, Joon Jeong, Kweon-Cheon Kim, Soo Youn Bae

**Affiliations:** ^1^Department of Surgery, Chungbuk National University Hospital, College of Medicine, Chungbuk National University, Cheongju, South Korea; ^2^Clinical Research Center, Asan Institute for Life Sciences, Asan Medical Center, University of Ulsan College of Medicine, Seoul, South Korea; ^3^Department of Biostatistics, Korea University, Seoul, South Korea; ^4^Advertising and Branding, Kaywon University of Art and Design, Uiwang-si, South Korea; ^5^Division of Breast Surgery, Department of Surgery, Yonsei University College of Medicine, Seoul, South Korea; ^6^Department of Surgery, Jeonbuk National University Medical School, Jeonju, South Korea; ^7^Department of Surgery, University of Ulsan College of Medicine, Asan Medical Center, Seoul, South Korea; ^8^Department of Food and Nutrition, College of Human Ecology, Seoul National University, Seoul, South Korea; ^9^Department of Surgery, Soonchunhyang University Seoul Hospital, Seoul, South Korea; ^10^Department of Surgery, National Health Insurance Service Ilsan Hospital, Koyang-si, South Korea; ^11^Department of Surgery, Gangnam Severance Hospital, Yonsei University College of Medicine, Seoul, South Korea; ^12^Department of Surgery, Chosun University Medical School, Gwangju, South Korea; ^13^Department of Surgery, Seoul St. Mary’s Hospital, The Catholic University of Korea, Seoul, South Korea

**Keywords:** breast neoplasm, postpartum, pregnancy, delivery, gestational, diabetes

## Abstract

The term ‘pregnancy-associated breast cancer’ is no longer used as it has been consistently reported that breast cancer during pregnancy and breast cancer after delivery (postpartum breast cancer) have different characteristics and prognosis. The purpose of this study is to define postpartum breast cancer by analyzing the incidence rate, related factors, and prognosis according to the timing of breast cancer. Data from the Korean National Health Insurance Service were used to analyze 1,292,727 women aged 20-49 years who birthed their first child between 2007 and 2012. The annual incidence rate of breast cancer after delivery increased every year (7.7 per 10,000 person-years after 5 years, 19.36 per 10,000 person-years after 10 years). The risk of breast cancer was significantly higher (hazard ratio 1.15, 95% CI 1.05-1.27, P=0.0037) in women diagnosed with gestational diabetes, but that was not associated with overall survival (OS). Patients diagnosed with breast cancer within 5 years of delivery had a poorer prognosis than those diagnosed later (5-year OS, <5 years: 91.1% vs. 5-10 years: 96.0%). In multivariate analysis of OS, the hazard ratio of patients diagnosed within 5 years after delivery was twice as high as of patients diagnosed between 5 and 10 years. Women diagnosed with gestational diabetes had an increased risk of breast cancer. Breast cancer patients diagnosed within 5 years of delivery had a poorer prognosis than those diagnosed later. In this regard, careful screening for early diagnosis of high-risk patients and intensive research on new treatment strategies are needed.

## Introduction

Pregnancy, childbirth and lactation produce the greatest physiologic changes in the breast. Breast cancer is related to the duration of exposure to female hormones, and it is well known that childbirth and breast feeding lower long-term risk of breast cancer. However, a temporary increase in the risk of breast cancer after pregnancy and childbirth has also been reported (1–3).

During pregnancy, the ductal system undergoes rapid proliferation and forms intra-branch alveolar structures extending from the ducts, which are used for milk production. The unique biological characteristics of the involution process, the process in which the breast returns to the mammary state before pregnancy after lactation, are related to the development of breast cancer after childbirth (3, 4). Similar to the wound healing process, immune cell influx during mammary gland involution is thought to be associated with tumor progression (5).

Studies of mouse mammary glands suggest that the involution process is also related to the induction of acute-phase response genes and increases in inflammatory cytokines and modulators of apoptosis and immune cascades (3, 6–8), and remodeling of the postpartum mammary microenvironment promotes tumor growth and tumor cell invasion and metastasis (9–11).

In the past, the term pregnancy-associated breast cancer (PABC) was used to describe breast cancer in relation to pregnancy and childbirth (12). PABC has various definitions, but is generally defined as breast cancer diagnosed during pregnancy or within 1 year after delivery. PABC has a poor prognosis due to late diagnosis or limitations in treatment.

However, more recently, the disease has been divided into breast cancer during pregnancy and breast cancer after delivery (postpartum breast cancer) (12, 13). Breast cancer during pregnancy can be treated with chemotherapy from the second trimester, and there are many reports that the prognosis is not different from that of non-PABC (12, 14–18). However, postpartum breast cancer accounts for more than 50% of breast cancers in young women and has a poorer prognosis than breast cancer in nulliparous women (19–22).

The definition of postpartum breast cancer is also diverse, with some definitions based on 1 year or 5 years postpartum, while recently an increase in risk has been reported up to 22 years (17, 18, 20, 22). In terms of risk of breast cancer, postpartum breast cancer is sometimes defined on the basis that the incidence rate temporarily increases for up to 5 years after delivery, and thereafter there is no difference from the incidence rate of the general population. In terms of prognosis, postpartum breast cancer is also defined on the basis that the prognosis of breast cancer that occurs within a certain period after delivery is different from that of breast cancer that occurs later (23).

Therefore, in this study, the following questions were explored to define and understand the characteristics of postpartum breast cancer.

1) Breast cancer incidence rate according to time after delivery - Is there a period when the incidence of breast cancer increases after delivery?
2) Breast cancer after delivery (postpartum breast cancer) risk factors and related diseases - Are there factors associated with increased incidence of breast cancer after delivery? (age, frequency, interval, comorbidities)
3) Clinical features and pathological characteristics according to the interval between delivery and breast cancer - Are there any differences in the characteristics of breast cancer according to onset timing after childbirth?
4) Prognosis according to the interval between delivery and breast cancer - Does breast cancer occurring within 5 years of delivery have a worse prognosis?

## Methods

In Korea, all medical providers and users (all citizens) are obliged to subscribe to the National Health Insurance Service (NHIS). The NHIS categorizes insurance qualifications and payment amount by insured person, and insurance policyholders (all citizens) are required to pay premiums to the NHIS. When medical services are used at a healthcare institution, the healthcare institution bills the Health Insurance Review and Assessment Service (HIRA) for the cost of medical care benefits, excluding out-of-pocket expenses. The NHIS pays insurance money, and the HIRA reviews insurance claims and evaluates the quality of medical services.

The NHIS includes data on eligibility and insurance premiums from birth to death, hospital and hospital usage history and national health checkup results, rare incurable disease and cancer registration information, medical benefit data, and long-term care data for the elderly. The National Health Insurance Sharing Service (https://nhiss.nhis.or.kr/bd/ay/bdaya001iv.do) had provided support of policy and academic research utilizing National Health information since 2002. Research data is largely provided as a customized DB and a sample cohort DB. “Customized DB” refers to data that are processed and provided as demand-tailored data so that the health information data that are collected, held, and managed by the NHIS can be used for policy and academic research purposes. Customized health information data are provided using statistical analysis tools in the “data analysis room,” which is a place in the industrial complex where a PC can be used for research and analysis because the size of the data is very large. The NHIS covers all citizens who reside in Korea except Medical Aid Beneficiaries and Health Care Beneficiaries for veterans; the NHIS covers 97% of the Korean population.

Among the data from 1,620,700 female patients provided by the NHIS, this study was conducted on those under the age of 50 who experienced their first childbirth between 2007 and 2012. Eighteen patients with the same date of birth and death were excluded. Finally, 1,292,706 patients were enrolled. Of these, 235,872 (18.25%) women were defined as having their first childbirth in 2007. The last follow-up of these subjects was December 31, 2017.

### Definition

Delivery was defined for inpatients using the Code of Conduct (ICD-9-CM Procedures Vol 3. version 32). First delivery was defined as a case where there was no insurance claim related to delivery between 2002 and 2006. Delivery included all kinds of delivery including vaginal delivery and Caesarean section, and the codes are listed in the **Supplement (S1)**. Breast cancer that occurred during the observation period was defined as ICD-10 codes C50 and D05, and cases with breast cancer codes before first delivery were excluded. Preeclampsia/eclampsia was defined as cases claimed under ICD-10 codes O11, O14, and O15. Gestational diabetes was defined based on code O24.

### Statistical Analyses

The baseline characteristics of the women with/without breast cancer were compared using Student’s t-test and the chi-square test. The annual incidence rates of breast cancer were calculated by Poisson regression. Characteristics of breast cancer occurring within 5 years/5 to 10 years/>10 years after delivery were compared and analyzed using ANOVA and chi-square test. For survival analysis, the Kaplan-Meier method and log rank test and Cox proportional hazards regression were used. The Cox proportional hazards model was used to estimate the hazard ratios (HR) and 95% confidence intervals (CIs) and to identify prognostic factors for survival. P-values < 0.05 were considered statistically significant. Statistical analyses were performed using Statistical Analysis System (SAS) version 9.4 (SAS Institute, Cary, NC, USA).

This study was approved by the Institutional Review Board of the Korea University Anam Hospital (No. 2020an0530).

## Results

### Baseline Characteristics

The median age of the 1,292,706 women who had their first delivery between 2007 and 2012 was 30 years (range 28-33 years), among whom 44.61% were in their 20s (20-29 years), 53.23% in their 30s (30-39 years), and 2.13% in their 40s (40-49 years) (**S2**). During the follow-up period, 67.6% of women gave birth once, 30.7% of women gave birth twice, and 1.7% of women gave birth three or more times. During the observation period, 11,927 women were diagnosed with breast cancer and the median age at diagnosis was 39 years. The first childbirth age of these patients was a median of 32 years, and 70.6% of patients gave birth between the ages of 30-39 years. The period from first delivery to breast cancer diagnosis was within 5 years in 23.5%, from 5 to 10 years in 57.8%, and over 10 years in 18.6%.

The median age of the 235,872 women who had their first delivery in 2007 was 30 years (range 27-32 years) (**Table 1**). In total, 48.97% of these women were in their 20s (20-29 years) for their first delivery, 49.34% in their 30s (30-39 years), and 1.69% in their 40s (40-49 years). During the 5 years from 2007 to 2012, 44.55% of these women experienced one delivery, 50.11% two deliveries, and 5.33% three or more deliveries. Among women who delivered twice, the interval between the two deliveries was 12-24 months in 24.73%, 24-36 months in 35.33%, 36-48 months in 12.85%, and 60 months or longer in 3.82%.

**Table 1:** Annual incidence rate of women who had first delivery (a) between 2007 and 2012 (b) in 2007

1) women who had first delivery between 2007 and 2012			
Follow-up duration (years)	No. of population	No. of event	Incidence rate per 10,000 person-years (95% CI)
0 – 1 yr	1292348	275	2.13 (1.88, 2.39)
1yr – 2yr	1291725	434	3.36 (3.05, 3.69)
2yr – 3yr	1290920	553	4.28 (3.93, 4.66)
3yr – 4yr	1289963	667	5.17 (4.79, 5.58)
4yr – 5yr	1288856	878	6.81 (6.37, 7.28)
5yr – 6yr	1287471	1138	8.84 (8.33, 9.37)
6yr – 7yr	1285796	1385	10.77 (10.21, 11.35)
7yr – 8yr	1178336	1575	13.37 (12.71, 14.04)
8yr – 9yr	967373	1437	14.85 (14.10, 15.64)
9yr – 10yr	755993	1359	17.98 (17.03, 18.96)
10yr – 11yr	553585	1166	21.06 (19.87, 22.31)
11yr – 12yr	342787	770	22.46 (20.90, 24.11)
>12yr	112901	290	25.69 (22.81, 28.82)

**2) women who had first delivery in 2007**			
**Follow-up duration (years)**	**No. of population**	**No. of event**	**Incidence rate per 10,000 person-years (95% CI)**
0 – 1 yr	235804	59	2.50 (1.90, 3.23)
1yr – 2yr	235702	70	2.97 (2.32, 3.75)
2yr – 3yr	235563	90	3.82 (3.07, 4.70)
3yr – 4yr	235388	120	5.10 (4.23, 6.10)
4yr – 5yr	235188	138	5.87 (4.93, 6.93)
5yr – 6yr	234939	181	7.70 (6.62, 8.91)
6yr – 7yr	234656	225	9.59 (8.38, 10.93)
7yr – 8yr	234350	223	9.52 (8.31, 10.85)
8yr – 9yr	234023	239	10.21 (8.96, 11.59)
9yr – 10yr	233650	361	15.45 (13.90, 17.13)
10yr – 11yr	233134	451	19.35 (17.60, 21.22)
11yr – 12yr	232548	524	22.53 (20.64, 24.55)
>12yr	112901	290	25.69 (22.81, 28.82)

*By poisson regression.

Of the 235,872 women who gave birth in 2007, 2,971 patients were diagnosed with breast cancer. The median age at the time of diagnosis of breast cancer in these patients was 40 years (33-44 years), 37.80% of patients were in their 30s (30-39 years) and 58.57% of patients were in their 40s (40-49 years). The median age at first delivery of these breast cancer patients was 32 years (29-35 years), of whom 28.58% were in their 20s (20-29 years) and 68.02% were in their 30s (30-39 years). There were 1,681 cases (56.58%) of single births, 1,194 cases (40.19%) of two births, and 96 cases (3.23%) of three or more births. Among the 1,194 breast cancer patients who delivered twice, the interval between the two deliveries was 12-24 months in 295 (24.71%) and 24-36 months in 421 (35.26%). The period from first delivery to the diagnosis of breast cancer was less than 5 years in 477 patients (16.06%), between 5 and 10 years after delivery in 1,229 (41.37%), and more than 10 years in 1,265 (42.58%).

### Incidence Rate of Breast Cancer

Among women who gave birth for the first time in 2007 and were observed for 12 years, the diagnosis of breast cancer showed a steady increase every year. The incidence of breast cancer 5 years after delivery was 7.7 per 10,000 person-years (95% CI 6.62-8.91), and this increased to 19.36 per 10,000 person-years after 10 years (95% CI 17.60-21.22) (**Table 1**). The incidence rate of breast cancer in women with a first delivery between 2007 and 2012 was 8.84 per 10,000 person-years (95% CI 8.33-9.37) after 5 years and 21.06 (95% CI 19.87-22.31) per 10,000 person-years after 10 years.

**Figure 1:**
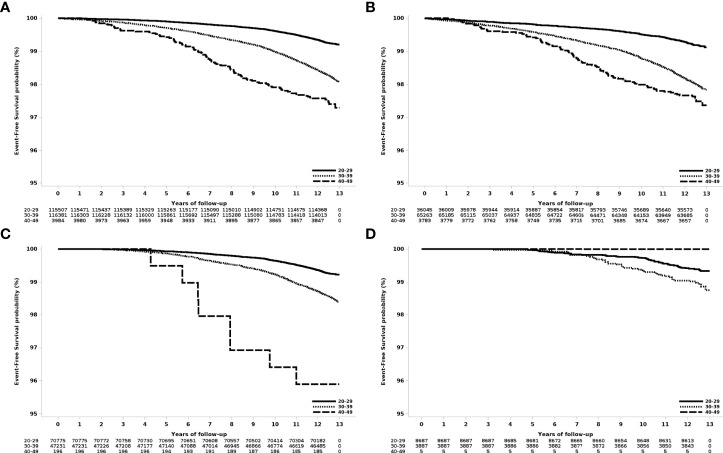
Event-free survival according to age at first delivery in women who gave birth in 2007. **(A)** All patients, **(B)** those with one delivery, **(C)** those with two deliveries, and **(D)** those with three or more deliveries.

The risk of breast cancer increased with age at first delivery (**Figure 1**). Compared to women who gave birth for the first time in their 20s, the risk of breast cancer was HR 2.36 (95% CI 2.18-.56), for women who gave birth for the first time in their 30s and HR 3.47 (2.82-4.26) for women who gave birth for the first time in their 40s (**Table 2**).

**Table 2:** Incidence rate of breast cancer in women who had first delivery in 2007.

	No. of patients(N = 235,872)	No. of Events(N = 2,971)	Incidence rate per 10,000 person years (95% CI)	10-Year cumulative rate	P-value by log rank test	Crude HR(95% CI)	P-value
Age at first delivery					<.0001		
<30	115507	849	5.92 (5.53-6.33)	0.39 (0.35-0.42)		Ref	
30-39	116381	2021	14.00 (13.40-14.62)	1.01 (0.95-1.07)		2.36 (2.18, 2.56)	<.0001
40-49	3984	101	20.56 (16.92-24.99)	2.09 (1.65-2.54)		3.47 (2.82, 4.26)	<.0001
Age at last delivery					<.0001		
20-29	66222	439	5.34 (4.86-5.86)	0.38 (0.34-0.43)		Ref	
30-39	163948	2396	11.77 (11.31-12.25)	0.82 (0.78-0.87)		2.20 (1.99, 2.44)	<.0001
40-49	5702	136	19.32 (16.33-22.86)	1.85 (1.50-2.20)		3.62 (2.99, 4.39)	<.0001
Number of delivery					<.0001		
1	105091	1681	12.92 (12.32-13.56)	1.00 (0.94-1.06)		Ref	
2	118202	1194	8.12 (7.67-8.60)	0.52 (0.48-0.56)		0.63 (0.58, 0.68)	<.0001
>=3	12579	96	6.12 (5.01-7.47)	0.39 (0.28-0.50)		0.47 (0.38, 0.58)	<.0001
Interval of delivery (women with two deliveries)					0.0973		
≥ 10 months, < 12 months	570	9	12.70 (6.61-24.42)	0.70 (0.02-1.39)		2.48 (1.18, 5.22)	0.0170
≥ 12 months, < 24 months	29231	295	8.12 (7.25-9.11)	0.58 (0.49-0.67)		1.59 (1.09, 2.31)	0.0159
≥ 24 months, < 36 months	41757	421	8.12 (7.38-8.93)	0.50 (0.44-0.57)		1.59 (1.10, 2.30)	0.0144
≥ 36 months, < 48months	26936	273	8.15 (7.23-9.17)	0.50 (0.42-0.59)		1.59 (1.09, 2.32)	0.0159
≥ 48 months, < 60 months	15187	166	8.78 (7.54-10.22)	0.51 (0.40-0.63)		1.71 (1.16, 2.53)	0.0066
≥ 60 months	4521	30	5.25 (3.67-7.51)	0.38 (0.20-0.55)		Ref	

The risk of breast cancer also increased with age at the last delivery. Compared to women in their 20s during their last delivery, the risk of breast cancer was HR 2.20 (95% CI 1.99-2.44) when their last delivery was in their 30s, and HR 3.62 (95% CI 2.99-4.39) when their last delivery was in their 40s.

**Figure 2:**
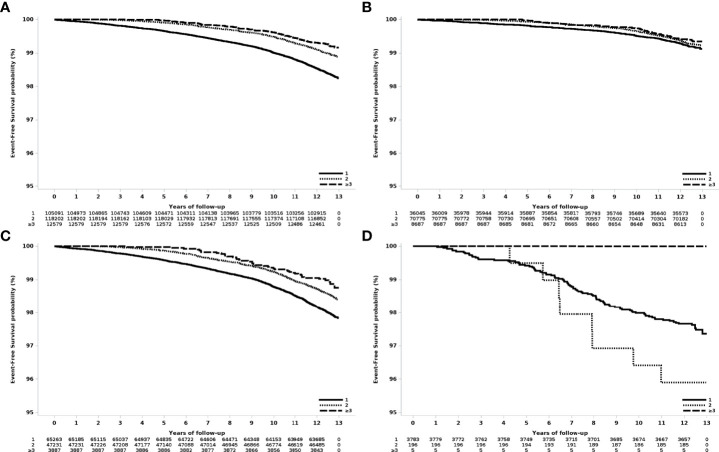
Event-free survival according to the number of deliveries in women who had their first delivery in 2007. **(A)** All patients, **(B)** age at first delivery: 20-29 years old, **(C)** age at first delivery 30-39 years old, and **(D)** age at first delivery 40-49 years old.

The risk of breast cancer decreased as the number of deliveries increased (**Figure 2**). Compared to women who delivered only once, women who delivered twice had a risk of HR 0.63 (95% CI 0.58-0.68), and those who delivered three or more times showed a risk of HR 0.47 (95% 0.38-0.58).

In women who delivered twice, the risk of breast cancer was significantly higher when the two deliveries were less than 60 months apart compared with more than 60 months apart (HR 1.59 to 2.48). The HR increased to 2.48 (95% CI 1.18-5.22) when the delivery interval was less than 12 months, compared with more than 60 months.

### Subgroup Analysis by Age at First Delivery and Number of Deliveries

Analysis of the risk of breast cancer by age at first delivery and number of deliveries revealed that risk decreased as the number of deliveries increased in women who gave birth in their 30s. The incidence rate per 10,000 person-years was 16.09 in women who delivered once, 11.55 in women who delivered twice, and 8.66 in women who delivered three or more times (**S3**). In women who gave birth in their 30s, the HR for women who delivered twice (0.72 [95% CI 0.65-0.79]) and who delivered three or more times (0.54 [95% CI 0.39-0.73]) were significantly lower compared to women who delivered once. Among women who delivered once or twice, those who gave first birth in their 30s and 40s had a significantly higher risk of breast cancer than those who gave birth for the first time in their 20s (**S4**).

### Pregnancy-Associated Diseases (Preeclampsia, Gestational Diabetes Mellitus)

During the observation period, 3,410 (1.45%) of 235,872 patients were diagnosed with preeclampsia, and 51 (1.49%) of these patients were also diagnosed with breast cancer. There was no significant difference in the risk of breast cancer with or without preeclampsia (HR 1.20, 95% CI 0.91-1.58*, P*=0.1953) (**Table 3**).

**Table 3:** Incidence rate by pregnancy associated diseases (a) preeclampsia, (b) gestational diabetes mellitus in women who had first delivery in 2007.

	No. of patients (N = 235,872)	No. of Events (N = 2,971))	Incidence rate per 10,000 person-years (95% CI)	10-Yearcumulative rate	p-value by log rank test	HR (95% CI)	p-value*
Preeclamsia					0.1950		
No	232,462	2,920	10.12 (9.76-10.49)	0.72 (0.68-0.75)		ref	
Yes	3,410	51	12.13 (9.22-15.96)	1.12 (0.77-1.48)		1.20 (0.91, 1.58)	0.1953
Gestational DM					0.0037		
No	200,242	2,467	9.92 (9.54-10.32)	0.71 (0.67-0.75)		ref	
Yes	35,630	504	11.41 (10.46-12.45)	0.80 (0.71-0.89)		1.15 (1.05, 1.27)	0.0037

*P-value by Cox PH regression.

During the observation period, 35,630 (15.11%) patients were diagnosed with gestational diabetes mellitus (GDM), and 504 (1.41%) of these patients were also diagnosed with breast cancer. The annual breast cancer incidence rate in women diagnosed with GDM continued to increase, and the incidence was higher than that of women who were not diagnosed with GDM. The annual incidence rate in women with GDM was 9.58 (95% CI 6.63-13.38) after 5 years and 30.48 (95% CI 24.98-36.83) after 11 years (**S5**). The risk of breast cancer was significantly higher (HR 1.15, 95% 1.05-1.27, P=0.0037) in women diagnosed with GDM than in women without GDM.

### Overall Survival

There were differences in overall survival according to age at diagnosis, age at first childbirth, treatment and timing of diagnosis of breast cancer after childbirth (**Table 4**). On univariate analysis, age at diagnosis of breast cancer, age at first and last delivery, and number of deliveries were not related to overall survival. However, there was a significant difference in overall survival according to the duration from first delivery to diagnosis of breast cancer and duration from last delivery to diagnosis of breast cancer. Compared to those diagnosed within 5 years of first delivery, HR were 0.44 (95% CI, 0.30-0.64, P <0.0001) and 0.42 (95% CI 0.24-0.71, P=0.0013) for those diagnosed 5-10 years or >10 years after first delivery, respectively (**Table 4**).

**Table 4:** Univariate analysis of survival (overall survival) for breast cancer patients.

	No of patient (n = 2971)	No of event (n = 145)	Incidence rate per 10,000 person-years (95% CI)	5-Year overall survival	10-Year overall survival	p-value*	Unadjusted HR (95% CI)	p-value**
Age at diagnosis of breast cancer						0.0391		
20-29	35	5	146.69 (61.06-352.44)	91.43 (82.15-100.00)	84.21 (71.34-97.08)		ref	
30-39	1123	87	129.06 (104.60-159.23)	92.64 (90.96-94.33)	88.80 (86.28-91.31)		0.73 (0.30, 1.81)	0.4988
40-49	1740	52	89.80 (68.43-117.84)	95.37 (93.97-96.77)	93.49 (90.82-96.17)		0.46 (0.18, 1.17)	0.1024
≥50	73	1	59.44 (8.37-422.00)	97.50 (92.66-100.00)	Non-estimable		0.30 (0.03, 2.55)	0.2680
Age at first delivery						0.404		
20-29	849	32	91.83 (64.94-129.85)	94.95 (92.95-96.95)	89.85 (84.92-94.78)		ref	
30-39	2021	107	119.37 (98.76-144.27)	93.36 (91.99-94.73)	90.41 (88.25-92.56)		1.31 (0.88, 1.94)	0.1850
40-49	101	6	101.27 (45.50-225.42)	96.80 (93.24-100.00)	88.75 (79.04-98.47)		1.13 (0.47, 2.69)	0.7909
Age at last delivery						0.7965		
20-29	439	19	94.77 (60.45-148.57)	95.23 (92.96-97.49)	92.30 (87.67-96.94)		ref	
30-39	2396	117	113.63 (94.80-136.20)	93.66 (92.39-94.93)	90.07 (87.83-92.30)		1.16 (0.72, 1.89)	0.5389
40-49	136	9	121.73 (63.34-233.95)	94.82 (90.75-98.90)	87.61 (78.47-96.75)		1.25 (0.57, 2.77)	0.5768
Number of delivery						0.5631		
1	1681	93	114.98 (93.83-140.89)	93.65 (92.22-95.07)	89.90 (87.54-92.27)		ref	
2	1194	47	102.35 (76.90-136.22)	94.42 (92.64-96.20)	91.01 (87.09-94.94)		0.84 (0.59, 1.19)	0.3220
>=3	96	5	138.77 (57.76-333.40)	94.05 (88.09-100.00)	86.82 (72.13-100.00)		1.13 (0.46, 2.78)	0.7894
Time-since-first-delivery						<.0001		
< 5yr	477	67	152.74 (120.21-194.06)	89.73 (87.00-92.45)	85.59 (82.37-88.80)		ref	
5yr-10yr	1229	57	89.78 (69.25-116.39)	95.26 (93.99-96.53)	Non-estimable		0.44 (0.30, 0.64)	<.0001
>=10yr	1265	21	91.09 (59.39-139.70)	Non-estimable	Non-estimable		0.42 (0.24, 0.71)	0.0013
Time-since-last delivery						<.0001		
< 5yr	759	87	143.17 (116.04-176.65)	91.10 (89.06-93.13)	87.16 (84.53-89.79)		ref	
5yr-10yr	1508	46	79.86 (59.82-106.62)	96.01 (94.80-97.23)	Non-estimable		0.44 (0.30, 0.64)	<.0001
>=10yr	704	12	99.62 (56.58-175.42)	Non-estimable	Non-estimable		0.53 (0.28, 1.01)	0.0546
Preeclampsia						0.5516		
No	2920	141	110.44 (93.64-130.26)	94.02 (92.93-95.11)	90.33 (88.31-92.36)		ref	
Yes	51	4	145.99 (54.79-388.97)	91.55 (82.11-100.00)	85.01 (69.87-100.00)		1.35 (0.50, 3.65)	0.5516
Gestational DM						0.5560		
No	2467	119	108.53 (90.68-129.89)	94.10 (92.91-95.28)	90.10 (87.87-92.33)		ref	
Yes	504	26	125.20 (85.25-183.89)	93.28 (90.55-96.02)	91.48 (88.13-94.84)		1.14 (0.74, 1.74)	0.5562

*P-value by log rank test.

**P-value by Cox PH regression.

In the analysis of overall survival adjusted for age at diagnosis of breast cancer, patients diagnosed within 5 years after their first delivery had a significantly poorer prognosis than those diagnosed later (diagnosed at 5-10 years, HR 0.48, 95% 0.32-0.71, P=0.002; diagnosed at ≥10 years, HR 0.49, 95% 0.27-0.87, P=0.0152) (**Table 5**). After adjustment for age and treatment method (endocrine therapy, chemotherapy, targeted therapy) at the time of breast cancer diagnosis, patients diagnosed within 5 years of delivery had a significantly worse prognosis than patients diagnosed 5 to 10 years after delivery (HR 0.50, 95% CI 0.34-0.74, P=0.0006)

**Table 5:** Multivariate analysis (model I) of survival (overall survival) for breast cancer patients in women who had first delivery in 2007.

		Adjusted Hazard Ratio (95% CI)	P Value
(model I)			
Age at diagnosis of breast cancer	20-29	ref	
	30-39	0.94 (0.38, 2.33)	0.8874
	40-49	0.73 (0.28, 1.91)	0.5270
	≥50	0.51 (0.06, 4.56)	0.5506
Time-since-first-delivery	<5yr	ref	
	5yr-10yr	0.48 (0.32, 0.71)	0.0002
	>10yr	0.49 (0.27, 0.87)	0.0152
(model II)			
Age at diagnosis of breast cancer	20-29	ref	
	30-39	0.73 (0.29, 1.83)	0.5029
	40-49	0.78 (0.30, 2.04)	0.6070
	≥50	0.42 (0.05, 3.71)	0.4324
Time -since-first-delivery	<5yr	ref	
	5yr-10yr	0.50 (0.34, 0.74)	0.0006
	>10yr	0.63 (0.35, 1.14)	0.1305
Endocrine therapy	No	ref	
	Yes	0.29 (0.20, 0.42)	<.0001
Chemotherapy	No	ref	
	Yes	6.59 (4.08, 10.65)	<.0001
Target therapy	No	ref	
	Yes	0.89 (0.57, 1.38)	0.6025

## Discussion

This study sought to determine whether there is transient increased risk of breast cancer after delivery. Previous studies reported an increased incidence of breast cancer 5–10 years after childbirth (13, 17, 21, 24). However, on follow-up for up to 12 years after delivery in this study, a continuous increase in the incidence of breast cancer was confirmed. The period during which an increase in the risk of breast cancer is observed after delivery may vary due to differences in study sample such as race, age range of subjects, and age at first childbirth. In this study, women under the age of 50 were defined and analyzed as women of childbearing age. Compared to other studies, this age range of subjects is relatively wide.

We also evaluated risk factors for breast cancer related to delivery. As previously established, the older women are at first delivery, the higher their risk of breast cancer is. Among women with one or two deliveries, risk increased with the age at first delivery. There was no difference according to age at first delivery when the number of deliveries was three or more. Higher risk of breast cancer was also observed with older age at last delivery. However, a higher the number of deliveries decreased risk of breast cancer. Even in the case of women whose first delivery occurred in their 30s, a decrease in risk of breast cancer was observed as the number of deliveries increased. However, in the case of women who delivered less than two times, the risk of breast cancer also appears to increase with age at first delivery. The risk of breast cancer was higher in cases with a delivery interval of less than 5 years than if the delivery interval was more than 5 years. Although the number of patients was small, breast cancer risk increased by HR 1.6-1.7 when the delivery interval was less than 1 year.

Preeclampsia is a multisystem syndrome that occurs in 2% to 5% of pregnancies (25). A previous systematic review and meta-analysis as well as prospective and retrospective cohort studies reported no increased risk of breast cancer (HR 1.04, 95% 0.78 to 1.39) due to preeclampsia (26). A population study also demonstrated no association between antiangiogenic factor levels during pregnancy and risk of breast cancer in the first decade after delivery (27). However, in a recently published meta-analysis that included 13 cohort studies comprising 5,254,150 participants, women with preeclampsia had a lower incidence of breast cancer than women without preeclampsia (28). One possible mechanism for this reduced risk of breast cancer is hormonal changes and responsiveness to hormones. Compared with healthy pregnant women, pregnant women with preeclampsia have low estrogen levels and high progesterone levels, and these hormonal changes may suppress estrogen-induced cancer (29, 30). Inflammatory responses and antiangiogenic factors during pregnancy may also be associated with breast cancer prevention or better prognosis (31, 32). However, there was no association between breast cancer and preeclampsia in this study. Preeclampsia/pregnancy hypertension does not increase the risk of breast cancer, although this may vary by race and age of the population.

In this study, GDM was identified as a risk factor for breast cancer. Several previous studies have also shown that diabetes mellitus (primarily type 2) is associated with increased risk of breast cancer (33–36). However, studies to date on GDM and breast cancer have shown conflicting results. There was no association with increased risk of GDM and invasive breast cancer in a large cohort of American women, the Nurses Health Study II (37), while the Sister Study (1,609 invasive breast cancers) reported a positive association between GDM and estrogen receptor (ER)-negative breast cancer (38). In a long-term cohort study of 753 women in New Zealand, gestational glucose intolerance was found to be associated with breast cancer (39). However, a US study of 1,239 women diagnosed with breast cancer and 1,166 controls did not find an association between GDM and breast cancer (40). With regard to menopause status, GDM was related to elevated risk of postmenopausal breast cancer (29) and reduced risk of premenopausal breast cancer (41). However, although GDM increased the risk of breast cancer, there was no difference in the survival rate of breast cancer patients according to GDM.

Whether postpartum breast cancer has a worse prognosis also remains unclear. PABC has a poorer prognosis than non-PABC. The definition of PABC varies, but it is generally defined as during pregnancy and within one year of delivery. A high frequency of higher grade, triple-negative breast cancers, and HER2+breast cancer was observed in PABC, and 5-year OS was worse (42–44). In a meta-analysis, PABC had a higher risk of death (OS) than non-PABC (HR 1.44, 95% CI 1.27-1.63) and postpartum breast cancer had poorer outcomes than breast cancer during pregnancy (45).

Recent studies have shown that breast cancer during pregnancy does not differ in prognosis from breast cancer in non-pregnant women. However, since postpartum breast cancer has a significantly poorer prognosis, it is more appropriate to divide breast cancer during pregnancy from postpartum breast cancer, rather than grouping them as PABC (13). In this study, patients diagnosed with breast cancer within 5 years postpartum had a poorer prognosis than those diagnosed >5 years postpartum. Since most patients diagnosed with breast cancer within 5 years of delivery are young women, the prognosis of breast cancer may be poor due to the characteristics of breast cancers that affect younger women. However, even after adjusting for age at diagnosis, prognosis was significantly poor in patients diagnosed with breast cancer within 5 years of delivery.

Breast cancer occurring within 5-10 years of delivery is estimated to account for 35-55% of all breast cancer cases in women under the age of 45 (20)[20]. Several previous studies of young breast cancer patients have shown that breast cancer within 5-10 years of delivery has a worse prognosis than that of breast cancer in nulliparous women (46–48). Many preclinical studies have suggested that it this related to a developmental tissue remodeling process of mammary gland involution (9–11, 13, 23, 49), which may result in distinct gene expression profiles. Recent studies have shown that postpartum breast cancer is a unique entity with distinct genomic signatures. Asztalos et al. reported that distinct immune signals persist for up to 10 years after delivery (50). Genomic alterations in breast cancer were associated with age at first pregnancy; the tumors that developed in early parous patients were characterized by a higher number of indels, a lower frequency of CDH1 mutations (1.2%), a higher frequency of TP53 mutations (50%), and MYC amplification (28%) (2). PABC was associated with increased tumor infiltrating lymphocyte (TIL) infiltration (2), and programmed cell death protein 1 (PD-L1) is highly expressed in TILs in PABC (51, 52). In a recent study using RNA expression data from clinically matched estrogen receptor positive (ER+) cases (n = 16), postpartum breast cancer had pronounced T-cell presence and T-cell activation/exhaustion signatures, reduced TP53 activity, reduced ER signaling, and increased cell cycle gene signatures compared with breast cancer in nulliparous women (53).

The major limitation of this study is that stage and subtype could not be confirmed from this data. However, when the pattern of adjuvant treatment was analyzed, patients diagnosed within 5 years had a higher proportion of chemotherapy than those diagnosed within 5-10 years (**S6**). This suggests that there are differences in stage and subtype between groups, and further research is needed.

In this study, breast cancer risk continued to increase steadily until 12 years after delivery and did not show an increase in a specific time period. Those diagnosed with GDM had a higher risk of breast cancer. Older age at first delivery, fewer total births, and shorter intervals between deliveries were associated with higher risk of breast cancer. However, there was no difference in OS based on these factors. For postpartum women with these risk factors, the importance of screening should be further emphasized. The prognosis of patients who developed breast cancer within 5 years after delivery was worse than that of patients diagnosed later. Therefore, intensive screening and new treatment strategies for postpartum breast cancer patients are necessary.

## Data Availability Statement

This data is managed by the National Health Insurance Service of Korea. Only approved researchers may use the data in the designated “data analysis room”. Requests to access these datasets should be directed to (https://nhiss.nhis.or.kr/bd/ay/bdaya001iv.do).

## Ethics Statement

This study was approved by the Institutional Review Board of the Korea University Anam Hospital (No. 2020an0530). Written informed consent for participation was not required for this study in accordance with the national legislation and the institutional requirements.

## Author Contributions

All authors contributed to the study conception and design. Material preparation, data collection and analysis were performed by SP, JSL, JY, and SYB. The first draft of the manuscript was written by SB and all authors commented on previous versions of the manuscript. All authors read and approved the final manuscript.

## Funding

This work was supported by the research grant of the Korean Breast Cancer Society.

## Conflict of Interest

The authors declare that the research was conducted in the absence of any commercial or financial relationships that could be construed as a potential conflict of interest.

## Publisher’s Note

All claims expressed in this article are solely those of the authors and do not necessarily represent those of their affiliated organizations, or those of the publisher, the editors and the reviewers. Any product that may be evaluated in this article, or claim that may be made by its manufacturer, is not guaranteed or endorsed by the publisher.
