# Supplementary material for: The Risk Factors, Incidence and Prognosis of Postpartum Breast Cancer: A Nationwide Study by the SMARTSHIP Group
Source: Front Oncol. 2022 Jul 1;12:889433. doi: 10.3389/fonc.2022.889433 (PMC9283825; doi:10.3389/fonc.2022.889433)
Supplement: Supplementary file 1 [file Table_1.docx]

S1. The code list of virginal delivery and Caesarean section

|  | ICD-10 codes | the Code of Conduct | |  |  |  |
| --- | --- | --- | --- | --- | --- | --- |
| Vaginal delivery | O80x, O757 | R4351R, R4353R, R4356R, R4358R, | | |  |  |
|  |  | R3131R, R3133R, R3136R, R3138R | | |  |  |
|  | O814 | R4362, R4362 / R3141R, R3143R, R3146R, R3148R | | | | |
|  |  | RA431R, RA432R, RA433R, RA434R | | |  |  |
|  |  | RA311R, RA312R, RA313R, RA314R | | |  |  |
|  |  | RA361R, RA362R / RA315R, RA316R, RA317R, RA318R | | | | |
|  |  |  |  |  |  |  |
| Caesarean section | O342, O82x, O842, O902 | R4516, R4516R, R4517, R4518, R4519, R4520, R4507, R4508, | | | | |
|  |  | R5001, R5002, R4509, R4510, R4522 | | | |  |

S2. Baseline characteristics of breast cancer in women who had first delivery in 2007

|  |  | Total, n(%)  (N= 2971) |
| --- | --- | --- |
| Age at diagnosis of breast cancer | median (range) | 40 (37-44) |
|  | Mean±SD | 40.5 ± 4.73 |
|  | 20-29 | 35 (1.18) |
|  | 30-39 | 1123 (37.80) |
|  | 40-49 | 1740 (58.57) |
|  | ≥50 | 73 (2.46) |
| Age at first delivery | median (range) | 32 (29-35) |
|  | Mean±SD | 31.97 ± 3.9 |
|  | 20-29 | 849 (28.58) |
|  | 30-39 | 2021 (68.02) |
|  | 40-49 | 101 (3.40) |
| Age at last delivery | median (range) | 33 (31-36) |
|  | Mean±SD | 33.24 ± 3.68 |
|  | 20-29 | 439 (14.78) |
|  | 30-39 | 2396 (80.65) |
|  | ≥40 | 136 (4.58) |
| Number of delivery | 1 | 1681 (56.58) |
|  | 2 | 1194 (40.19) |
|  | >=3 | 96 (3.23) |
| Time time-since-first-delivery | < 5yr | 477 (16.06) |
|  | 5yr-10yr | 1229 (41.37) |
|  | >=10yr | 1265 (42.58) |
| Time time-since-last delivery | < 5yr | 759 (25.55) |
|  | 5yr-10yr | 1508 (50.76) |
|  | >=10yr | 704 (23.70) |
| Preeclampsia | No | 2920 (98.28) |
|  | Yes | 51 (1.72) |
| Gestational DM | No | 2467 (83.04) |
|  | Yes | 504 (16.96) |

S3.

S3. Baseline characteristics of breast cancer in women who had first delivery between 2007 and 2012

|  |  | Total, n(%)  (N=11,927) |
| --- | --- | --- |
| Age at diagnosis of breast cancer | median (range) | 39 (36-42) |
|  | Mean±SD | 39.47 ± 4.65 |
|  | 20-29 | 181 (1.52) |
|  | 30-39 | 5787 (48.52) |
|  | 40-49 | 5771 (48.39) |
|  | ≥50 | 188 (1.58) |
| Age at first delivery | median (range) | 32 (30-35) |
|  | Mean±SD | 32.34 ± 3.97 |
|  | 20-29 | 2915 (24.44) |
|  | 30-39 | 8420 (70.60) |
|  | 40-49 | 592 (4.96) |
| Time time-since-first-delivery | < 5yr | 2807 (23.53) |
|  | 5yr-9yr | 6894 (57.80) |
|  | >=10yr | 2226 (18.66) |
| Preeclampsia | No | 11714 (98.21) |
|  | Yes | 213 (1.79) |
| Gestational DM | No | 9773 (81.94) |
|  | Yes | 2154 (18.06) |

S4. Incidence rate of breast cancer by the age of first delivery in women who had first delivery in 2007

|  | No. of patients  (N=235,872) | No. of Events (N=2,971)) | Incidence rate  per 10,000 person-years (95% CI) | 10-Year cumulative rate | p-value* | HR (95% CI) |
| --- | --- | --- | --- | --- | --- | --- |
|  | 235,872 | 2,971 | 10.15 (9.79-10.52) | 0.72 (0.69-0.76) |  |  |
|  |  |  |  |  |  |  |
| Age at first delivery: 20-29 | 115,507 | 849 | 5.92 (5.53-6.33) | 0.39 (0.35-0.42) |  |  |
| Number of delivery |  |  |  |  | 0.1197 |  |
| 1 | 36,045 | 288 | 6.45 (5.75-7.24) | 0.49 (0.42-0.56) |  | 1(ref) |
| 2 | 70,775 | 507 | 5.76 (5.28-6.29) | 0.35 (0.31-0.39) |  | 0.89 (0.77, 1.03) |
| >=3 | 8,687 | 54 | 4.99 (3.82-6.51) | 0.27 (0.16-0.37) |  | 0.77 (0.58, 1.03) |
|  |  |  |  |  |  |  |
| Age at first delivery: 30-39 | 116,381 | 2,021 | 14.00 (13.40-14.62) | 1.01 (0.95-1.07) |  |  |
| Number of delivery |  |  |  |  | <.0001 |  |
| 1 | 65,263 | 1,300 | 16.09 (15.24-16.99) | 1.21 (1.13-1.30) |  | 1(ref) |
| 2 | 47,231 | 679 | 11.55 (10.71-12.45) | 0.76 (0.68-0.84) |  | 0.72 (0.65, 0.79) |
| >=3 | 3,887 | 42 | 8.66 (6.40-11.71) | 0.67 (0.41-0.93) |  | 0.54 (0.39, 0.73) |
|  |  |  |  |  |  |  |
| Age at first delivery: 40-49 | 3,984 | 101 | 20.56 (16.92-24.99) | 2.09 (1.65-2.54) |  |  |
| Number of delivery |  |  |  |  | 0.3502 |  |
| 1 | 3,783 | 93 | 19.93 (16.27-24.43) | 2.02 (1.57-2.47) |  | 1(ref) |
| 2 | 196 | 8 | 33.29 (16.65-66.57) | 3.59 (0.98-6.21) |  | 1.76 (0.87, 3.57) |
| >=3 | 5 | 0 | 0 | 0 |  | 3.97 (0.24, 65.30) |
|  |  |  |  |  |  |  |

S5. Incidence rate of breast cancer by the number of delivery in women who had first delivery in 2007

|  | No of patient | No of event | Incidence rate  per 10,000 person-years (95% CI) | 10-Year cumulative rate | p-value* | HR (95% CI) | p-value** |
| --- | --- | --- | --- | --- | --- | --- | --- |
|  | 235,872 | 2,971 | 10.15 (9.79-10.52) | 0.72 (0.69-0.76) |  |  |  |
|  |  |  |  |  |  |  |  |
| Number of delivery : 1 | 105,091 | 1,681 | 12.92 (12.32-13.56) | 1.00 (0.94-1.06) |  |  |  |
| Age at first delivery |  |  |  |  | <.0001 |  |  |
| 20-29 | 36,045 | 288 | 6.45 (5.75-7.24) | 0.49 (0.42-0.56) |  | ref |  |
| 30-39 | 65,263 | 1,300 | 16.09 (15.24-16.99) | 1.21 (1.13-1.30) |  | 2.49 (2.19, 2.83) | <.0001 |
| 40-49 | 3,783 | 93 | 19.93 (16.27-24.43) | 2.02 (1.57-2.47) |  | 3.08 (2.44, 3.89) | <.0001 |
|  |  |  |  |  |  |  |  |
| Number of delivery : 2 | 118,202 | 1,194 | 8.12 (7.67-8.60) | 0.52 (0.48-0.56) |  |  |  |
| Age at first delivery |  |  |  |  | <.0001 |  |  |
| 20-29 | 70,775 | 507 | 5.76 (5.28-6.29) | 0.35 (0.31-0.39) |  | ref |  |
| 30-39 | 47,231 | 679 | 11.55 (10.71-12.45) | 0.76 (0.68-0.84) |  | 2.00 (1.78, 2.24) | <.0001 |
| 40-49 | 196 | 8 | 33.29 (16.65-66.57) | 3.59 (0.98-6.21) |  | 5.80 (2.89, 11.67) | <.0001 |
|  |  |  |  |  |  |  |  |
| Number of delivery : 3 | 12,335 | 94 | 6.11 (4.99-7.48) | 0.39 (0.28-0.50) |  |  |  |
| Age at first delivery |  |  |  |  | 0.0329 |  |  |
| 20-29 | 8,500 | 53 | 5.00 (3.82-6.55) | 0.27 (0.16-0.38) |  | ref |  |
| 30-39 | 3,830 | 41 | 8.58 (6.32-11.65) | 0.65 (0.40-0.91) |  | 1.71 (1.14, 2.58) | 0.0099 |
| 40-49 | 5 | 0 | 0 | 0 |  | 15.76 (0.95, 260.65) | 0.0541 |
|  |  |  |  |  |  |  |  |
| Number of delivery : ≥3 | 12,579 | 96 | 6.12 (5.01-7.47) | 0.39 (0.28-0.50) |  |  |  |
| Age at first delivery |  |  |  |  | 0.0258 |  |  |
| 20-29 | 8,687 | 54 | 4.99 (3.82-6.51) | 0.27 (0.16-0.37) |  | ref |  |
| 30-39 | 3,887 | 42 | 8.66 (6.40-11.71) | 0.67 (0.41-0.93) |  | 1.73 (1.16, 2.60) | 0.0076 |
| 40-49 | 5 | 0 | 0 | 0 |  | 15.81 (0.96, 261.36) | 0.0538 |
| * By log rank test |  |  |  |  |  |  |  |
| ** By Cox's PH regression model | |  |  |  |  |  |  |

S6. Patients characteristics with breast cancer according to postpartum time in women who had first delivery in 2007

|  |  | Postpartum (Time-since-first-delivery), n(%) | | | |  |
| --- | --- | --- | --- | --- | --- | --- |
|  | Total, n(%) | <5yr | 5-10yr | >=10yr | | P-value |
|  | (N=2971) | (N=477) | (N=1229) | (N=1265) | |  |
| Age at diagnosis of breast cancer |  |  |  |  | |  |
| median (range) | 40 (37-44) | 35 (33-38) | 40 (37-43) | 42 (40-45) | |  |
| Mean±SD | 40.5 ± 4.73 | 35.54 ± 4.42 | 40.05 ± 4.14 | 42.80 ± 3.71 | | <.0001 (b) |
| 20-29 | 35 (1.18) | 30 (6.29) | 5 (0.41) | 0 (0.00) | | <.0001 (a) |
| 30-39 | 1123 (37.80) | 356 (74.63) | 553 (45.00) | 214 (16.92) | |  |
| 40-49 | 1740 (58.57) | 91 (19.08) | 654 (53.21) | 995 (78.66) | |  |
| ≥50 | 73 (2.46) | 0 (0.00) | 17 (1.38) | 56 (4.43) | |  |
| Age at first delivery |  |  |  |  | |  |
| median (range) | 32 (29-35) | 32 (30-35) | 32 (29-35) | 31 (29-34) | |  |
| Mean±SD | 31.97 ± 3.9 | 32.54 ± 4.22 | 32.19 ± 3.99 | 31.53 ± 3.64 | | <.0001 (b) |
| 20-29 | 849 (28.58) | 115 (24.11) | 333 (27.10) | 401 (31.70) | | <.0001 (a) |
| 30-39 | 2021 (68.02) | 339 (71.07) | 836 (68.02) | 846 (66.88) | |  |
| 40-49 | 101 (3.40) | 23 (4.82) | 60 (4.88) | 18 (1.42) | |  |
|  |  |  |  |  | |  |
| Endocrine therapy |  |  |  |  | | <.0001 (a) |
| No | 1256 (42.28) | 242 (50.73) | 389 (31.65) | 625 (49.41) | |  |
| Yes | 1715 (57.72) | 235 (49.27) | 840 (68.35) | 640 (50.59) | |  |
| Chemotherapy |  |  |  |  | | <.0001 (a) |
| No | 1572 (52.91) | 212 (44.44) | 512 (41.66) | 848 (67.04) | |  |
| Yes | 1399 (47.09) | 265 (55.56) | 717 (58.34) | 417 (32.96) | |  |
| Target therapy |  |  |  |  | | <.0001 (a) |
| No | 2641 (88.89) | 435 (91.19) | 1048 (85.27) | 1158 (91.54) | |  |
| Yes | 330 (11.11) | 42 (8.81) | 181 (14.73) | 107 (8.46) | |  |
|  |  |  |  |  | |  |
| Preeclampsia |  |  |  |  | | 0.0087 (a) |
| No | 2920 (98.28) | 462 (96.86) | 1206 (98.13) | 1252 (98.97) | |  |
| Yes | 51 (1.72) | 15 (3.14) | 23 (1.87) | 13 (1.03) | |  |
| Gestational DM |  |  |  |  | | 0.0247 (a) |
| No | 2467 (83.04) | 416 (87.21) | 1005 (81.77) | 1046 (82.69) | |  |
| Yes | 504 (16.96) | 61 (12.79) | 224 (18.23) | 219 (17.31) | |  |
| P-value by Chi-square test(a) and ANOVA(b) | | |  |  |  |  |

S7. Patients characteristics with breast cancer according to postpartum time in women who had first delivery between 2007 and 2012

|  |  | Postpartum , n(%) | | | P-value |
| --- | --- | --- | --- | --- | --- |
|  | Total, n(%) | <5yr | 5-10yr | >=10yr |  |
|  | (N=11927) | (N=2807) | (N=6894) | (N=2226) |  |
| Age at diagnosis of breast cancer |  |  |  |  |  |
| median (range) | 39 (36-42) | 35 (33-39) | 40 (37-42) | 42 (40-45) |  |
| Mean±SD | 39.47 ± 4.65 | 35.94 ± 4.42 | 39.89 ± 4.08 | 42.62 ± 3.70 | <.0001 (b) |
| Age at diagnosis of breast cancer |  |  |  |  | <.0001 (a) |
| 20-29 | 181 (1.52) | 151 (5.38) | 30 (0.44) | 0 (0.00) |  |
| 30-39 | 5787 (48.52) | **2030 (72.32)** | **3351 (48.61)** | 406 (18.24) |  |
| 40-49 | 5771 (48.39) | 624 (22.23) | **3419 (49.59)** | **1728 (77.63)** |  |
| ≥50 | 188 (1.58) | 2 (0.07) | 94 (1.36) | 92 (4.13) |  |
|  |  |  |  |  |  |
| Age at first delivery |  |  |  |  |  |
| median (range) | 32 (30-35) | 32 (30-36) | 32 (30-35) | 31 (29-34) |  |
| Mean±SD | 32.34 ± 3.97 | 32.89 ± 4.17 | 32.34 ± 3.95 | 31.68 ± 3.64 | <.0001 (b) |
| Age at first delivery |  |  |  |  | <.0001 (a) |
| 20-29 | 2915 (24.44) | 584 (20.81) | 1673 (24.27) | 658 (29.56) |  |
| 30-39 | 8420 (70.60) | 2020 (71.96) | 4879 (70.77) | 1521 (68.33) |  |
| 40-49 | 592 (4.96) | 203 (7.23) | 342 (4.96) | 47 (2.11) |  |
|  |  |  |  |  |  |
| Radiotherapy |  |  |  |  | <.0001 (a) |
| No | 5942 (49.82) | 1129 (40.22) | 3427 (49.71) | 1386 (62.26) |  |
| Yes | 5985 (50.18) | 1678 (59.78) | 3467 (50.29) | 840 (37.74) |  |
| Endocrine therapy |  |  |  |  | <.0001 (a) |
| No | 5459 (45.77) | 1286 (45.81) | 2980 (43.23) | 1193 (53.59) |  |
| Yes | 6468 (54.23) | 1521 (54.19) | 3914 (56.77) | 1033 (46.41) |  |
| Chemotherapy |  |  |  |  | <.0001 (a) |
| No | 6313 (52.93) | 1087 (38.72) | 3727 (54.06) | 1499 (67.34) |  |
| Yes | 5614 (47.07) | 1720 (61.28) | 3167 (45.94) | 727 (32.66) |  |
| Target therapy |  |  |  |  | <.0001 (a) |
| No | 10521 (88.21) | 2419 (86.18) | 6059 (87.89) | 2043 (91.78) |  |
| Yes | 1406 (11.79) | 388 (13.82) | 835 (12.11) | 183 (8.22) |  |
| Preeclampsia |  |  |  |  | 0.1294 (a) |
| No | 11714 (98.21) | 2747 (97.86) | 6785 (98.42) | 2182 (98.02) |  |
| Yes | 213 (1.79) | 60 (2.14) | 109 (1.58) | 44 (1.98) |  |
| Gestational DM |  |  |  |  | <.0001 (a) |
| No | 9773 (81.94) | 2198 (78.30) | 5575 (80.87) | 2000 (89.85) |  |
| Yes | 2154 (18.06) | 609 (21.70) | 1319 (19.13) | 226 (10.15) |  |
| Types of treatment |  |  |  |  | <.0001 (a) |
| No chemotherapy, target therapy, endocrine therapy | 3254 (27.28) | 560 (19.95) | 1840 (26.69) | 854 (38.36) |  |
| only endocrine therapy | 2999 (25.14) | 517 (18.42) | 1851 (26.85) | 631 (28.35) |  |
| only chemotherapy | 1634 (13.70) | 557 (19.84) | 816 (11.84) | 261 (11.73) |  |
| only target therapy | 17 (0.14) | 6 (0.21) | 11 (0.16) | 0 (0.00) |  |
| endocrine therapy + chemotherapy | 2634 (22.08) | 785 (27.97) | 1552 (22.51) | 297 (13.34) |  |
| target therapy + chemotherapy | 554 (4.64) | 163 (5.81) | 313 (4.54) | 78 (3.50) |  |
| endocrine therapy + target therapy | 43 (0.36) | 4 (0.14) | 25 (0.36) | 14 (0.63) |  |
| endocrine therapy+ chemotherapy+target therapy | 792 (6.64) | 215 (7.66) | 486 (7.05) | 91 (4.09) |  |
| P-value by Chi-square test(a) and ANOVA(b) | | | | | |

S8. Univariate analysis of survival (breast cancer specific survival or overall survival) for breast cancer patients (in women who had first delivery between 2007 and 2012)

|  | No of patient | No of event | Incidence rate | 5-Year overall survival | 10-Year overall survival | p-value* | Unadjusted HR (95% CI) | p-value** |
| --- | --- | --- | --- | --- | --- | --- | --- | --- |
|  |  |  | per 10,000 person-years (95% CI) |  |  |  |  |  |
| Age at diagnosis of breast cancer | |  |  |  |  | <.0001 |  |  |
| 20-29 | 181 | 22 | 161.63 (106.42-245.47) | 90.23 (85.81-94.65) | 85.56 (79.56-91.56) |  | ref |  |
| 30-39 | 5787 | 354 | 134.38 (121.09-149.14) | 92.94 (92.12-93.76) | 88.07 (86.50-89.64) |  | 0.77 (0.50, 1.19) | 0.2429 |
| 40-49 | 5771 | 159 | 91.26 (78.13-106.61) | 95.27 (94.42-96.12) | 92.61 (90.70-94.51) |  | 0.52 (0.33, 0.82) | 0.0051 |
| ≥50 | 188 | 8 | 203.54 (101.79-407.00) | 93.70 (89.36-98.04) | Non-estimable |  | 1.22 (0.54, 2.76) | 0.6286 |
| Age at first delivery |  |  |  |  |  | 0.7867 |  |  |
| 20-29 | 2915 | 128 | 120.27 (101.14-143.02) | 93.70 (92.44-94.96) | 88.93 (86.20-91.66) |  | ref |  |
| 30-39 | 8420 | 380 | 117.77 (106.51-130.23) | 93.69 (92.97-94.40) | 89.36 (87.83-90.88) |  | 0.97 (0.80, 1.19) | 0.7827 |
| 40-49 | 592 | 35 | 134.03 (96.23-186.68) | 94.11 (91.92-96.30) | 89.12 (84.66-93.57) |  | 1.09 (0.75, 1.59) | 0.6375 |
| Time time-since-first-delivery | |  |  |  |  | <.0001 |  |  |
| < 5yr | 2807 | 311 | 158.57 (141.89-177.22) | 91.32 (90.26-92.38) | 86.54 (84.99-88.10) |  | ref |  |
| 5yr-9yr | 6894 | 206 | 92.30 (80.52-105.81) | 95.39 (94.67-96.11) | Non-estimable |  | 0.52 (0.43, 0.63) | <.0001 |
| >=10yr | 2226 | 26 | 72.44 (49.32-106.40) | Non-estimable | Non-estimable |  | 0.45 (0.30, 0.68) | 0.0002 |
| Radiotherapy |  |  |  |  |  | 0.8377 |  |  |
| No | 5942 | 229 | 115.30 (101.29-131.24) | 93.91 (92.99-94.83) | 90.02 (88.30-91.74) |  | ref |  |
| Yes | 5985 | 314 | 122.38 (109.57-136.70) | 93.65 (92.87-94.44) | 88.54 (86.58-90.51) |  | 1.02 (0.86, 1.21) | 0.8379 |
| Endocrine therapy |  |  |  |  |  | <.0001 |  |  |
| No | 5459 | 364 | 189.48 (170.98-209.98) | 89.88 (88.75-91.00) | 85.96 (84.21-87.72) |  | ref |  |
| Yes | 6468 | 179 | 68.04 (58.77-78.77) | 96.43 (95.79-97.06) | 91.19 (89.09-93.30) |  | 0.34 (0.29, 0.41) | <.0001 |
| Chemotherapy |  |  |  |  |  | <.0001 |  |  |
| No | 6313 | 83 | 39.28 (31.67-48.70) | 97.86 (97.32-98.40) | 96.71 (95.51-97.91) |  | ref |  |
| Yes | 5614 | 460 | 188.63 (172.16-206.68) | 90.52 (89.57-91.46) | 83.89 (81.88-85.91) |  | 4.69 (3.71, 5.93) | <.0001 |
| Target therapy |  |  |  |  |  | 0.0011 |  |  |
| No | 10521 | 446 | 112.38 (102.42-123.30) | 94.23 (93.61-94.84) | 89.62 (88.24-91.00) |  | ref |  |
| Yes | 1406 | 97 | 166.37 (136.34-203.00) | 90.76 (88.81-92.72) | 87.71 (85.07-90.35) |  | 1.44 (1.15, 1.79) | 0.0012 |
| Preeclampsia |  |  |  |  |  | 0.4927 |  |  |
| No | 11714 | 535 | 119.81 (110.08-130.41) | 93.72 (93.12-94.32) | 89.23 (87.93-90.53) |  | ref |  |
| Yes | 213 | 8 | 92.40 (46.21-184.77) | 94.36 (89.74-98.99) | 90.03 (82.46-97.59) |  | 0.78 (0.39, 1.58) | 0.4943 |
| Gestational DM |  |  |  |  |  | 0.4276 |  |  |
| No | 9773 | 456 | 121.00 (110.39-132.63) | 93.80 (93.15-94.44) | 89.03 (87.60-90.45) |  | ref |  |
| Yes | 2154 | 87 | 111.08 (90.03-137.06) | 93.37 (91.83-94.92) | 90.90 (88.65-93.15) |  | 0.91 (0.72, 1.15) | 0.4278 |
| Types of treatment |  |  |  |  |  | <.0001 |  |  |
| No chemotherapy, target therapy, endocrine therapy | 3254 | 62 | 60.62 (47.26-77.75) | 96.78 (95.84-97.73) | 95.93 (94.40-97.47) |  | ref |  |
| only endocrine therapy | 2999 | 18 | 16.80 (10.58-26.66) | 99.02 (98.49-99.56) | 97.49 (95.50-99.48) |  | 0.26 (0.15, 0.44) | <.0001 |
| only chemotherapy | 1634 | 231 | 344.79 (303.08-392.25) | 82.90 (80.69-85.11) | 76.24 (72.84-79.65) |  | 5.54 (4.18, 7.34) | <.0001 |
| only target therapy | 17 | 1 | 124.94 (17.60-886.99) | 94.12 (82.93-100.00) | 94.12 (82.93-100.00) |  | 1.88 (0.26, 13.58) | 0.5306 |
| endocrine therapy + chemotherapy | 2634 | 135 | 112.09 (94.69-132.68) | 94.50 (93.38-95.62) | 86.55 (83.18-89.93) |  | 1.74 (1.29, 2.35) | 0.0003 |
| target therapy + chemotherapy | 554 | 70 | 317.75 (251.39-401.63) | 82.53 (78.40-86.67) | 78.05 (72.89-83.22) |  | 4.96 (3.52, 6.98) | <.0001 |
| endocrine therapy + target therapy | 43 | 2 | 184.47 (46.14-737.61) | 80.13 (50.80-100.00) | Non-estimable |  | 2.88 (0.70, 11.79) | 0.1409 |
| endocrine therapy+ chemotherapy+target therapy | 792 | 24 | 69.79 (46.78-104.12) | 96.19 (94.49-97.90) | 94.23 (91.68-96.78) |  | 1.07 (0.67, 1.71) | 0.7864 |
| * P-value by log rank test | |  |  |  |  |  |  |  |
| ** P-value by Cox PH regression | |  |  |  |  |  |  |  |

S9. multivariate analysis (model I)of survial (breast cancer specific survival or overall survival) for breast cancer patients

Model I

|  | Adjusted Hazard Ratio (95% CI) | P Value |
| --- | --- | --- |
| Age at first delivery |  |  |
| 20-29 | ref |  |
| 30-39 | 0.95 (0.77, 1.16) | 0.5837 |
| 40-49 | 1.00 (0.69, 1.46) | 0.9859 |
| Time-since-first-delivery |  |  |
| <5yr | ref |  |
| 5yr-9yr | 0.52 (0.43, 0.63) | <.0001 |
| >10yr | 0.45 (0.30, 0.68) | 0.0002 |

(Model II)

|  | Adjusted Hazard Ratio (95% CI) | P Value |
| --- | --- | --- |
| Age at first delivery |  |  |
| 20-29 | ref |  |
| 30-39 | 1.07 (0.87, 1.31) | 0.5147 |
| 40-49 | 1.26 (0.87, 1.84) | 0.2251 |
| Time-since-first-delivery |  |  |
| <5yr | ref |  |
| 5yr-9yr | 0.65 (0.54, 0.78) | <.0001 |
| >10yr | 0.66 (0.43, 1.00) | 0.0482 |
| Endocrine therapy |  |  |
| No | ref |  |
| Yes | 0.30 (0.25, 0.36) | <.0001 |
| Chemotherapy |  |  |
| No | ref |  |
| Yes | 5.39 (4.23, 6.85) | <.0001 |
| Target therapy |  |  |
| No | ref |  |
| Yes | 0.84 (0.67, 1.06) | 0.1366 |
